# The rapidly evolving view of lysosomal storage diseases

**DOI:** 10.15252/emmm.202012836

**Published:** 2021-01-18

**Authors:** Giancarlo Parenti, Diego L Medina, Andrea Ballabio

**Affiliations:** ^1^ Telethon Institute of Genetics and Medicine Pozzuoli Italy; ^2^ Department of Translational Medical Sciences Section of Pediatrics Federico II University Naples Italy; ^3^ Department of Molecular and Human Genetics Baylor College of Medicine Houston TX USA; ^4^ Jan and Dan Duncan Neurological Research Institute Texas Children Hospital Houston TX USA; ^5^ SSM School for Advanced Studies Federico II University Naples Italy

**Keywords:** autophagy, lysosomal biology, lysosomal storage diseases, lysosomes, Autophagy & Cell Death, Genetics, Gene Therapy & Genetic Disease

## Abstract

Lysosomal storage diseases are a group of metabolic disorders caused by deficiencies of several components of lysosomal function. Most commonly affected are lysosomal hydrolases, which are involved in the breakdown and recycling of a variety of complex molecules and cellular structures. The understanding of lysosomal biology has progressively improved over time. Lysosomes are no longer viewed as organelles exclusively involved in catabolic pathways, but rather as highly dynamic elements of the autophagic‐lysosomal pathway, involved in multiple cellular functions, including signaling, and able to adapt to environmental stimuli. This refined vision of lysosomes has substantially impacted on our understanding of the pathophysiology of lysosomal disorders. It is now clear that substrate accumulation triggers complex pathogenetic cascades that are responsible for disease pathology, such as aberrant vesicle trafficking, impairment of autophagy, dysregulation of signaling pathways, abnormalities of calcium homeostasis, and mitochondrial dysfunction. Novel technologies, in most cases based on high‐throughput approaches, have significantly contributed to the characterization of lysosomal biology or lysosomal dysfunction and have the potential to facilitate diagnostic processes, and to enable the identification of new therapeutic targets.

## Graphical abstract

In the last decade, our understanding of lysosomal storage diseases immensely improved. The current article comprehensively reviews recent advances in lysosomal storage diseases research and therapy development.

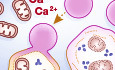

**Glossary**
Autophagy: A multistep and regulated pathway that removes unnecessary or dysfunctional cellular components and allows for delivery of cargo materials to lysosomes, where they are degraded and recycled.
High‐content imaging: Cell‐based technologies based on automated microscopy and complex image algorithms to extract multidimensional information on cell morphology, fluorescence intensity, or distribution of fluorescent markers within cells.
Genome editing and CRISPR‐Cas9: An RNA‐guided targeted genome editing tool: This methodology which allows to introduce different specific genetic changes such as gene knock‐out, knock‐in, insertions, and deletions in cell lines and in vivo.
Lysosomes: Membrane‐limited, ubiquitous, intracellular organelles involved in multiple cellular processes, such as catabolism and recycling of complex molecules and cellular components, signaling, and adaptation to environmental stimuli.
Metabolome analyses: High‐throughput methodologies for the detection of multiple metabolites, mainly based on mass spectrometry or nuclear magnetic resonance‐based approaches.
Next‐generation sequencing: A diagnostic tool based on technological platforms that allow for sequencing of millions of small fragments of DNA in parallel. Next‐generation sequencing can be used either for ”targeted” sequencing of selected gene panels or for “untargeted” approaches based on whole‐exome or genome analysis.
MicroRNAs: Small non‐coding RNAs that that regulate gene expression by targeting messenger RNAs.

## Introduction

**Figure 1:**
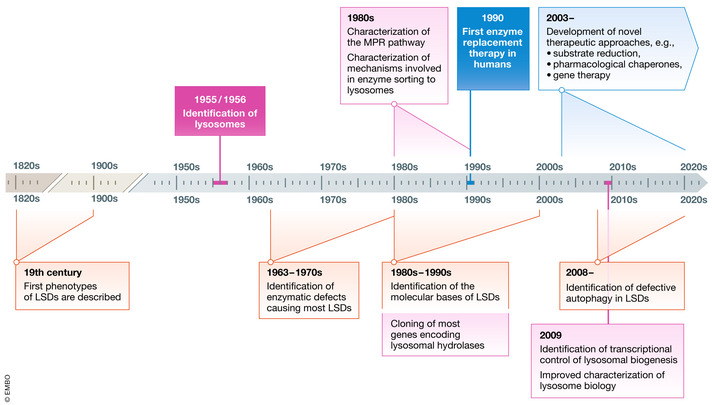
The evolution of the knowledge on LSDs After the identification of the first clinical phenotypes during of the 19th century, the knowledge on LSDs evolved following the recognition of lysosomes in 1955/56 and the demonstration of the biochemical defects underlying LSDs, starting from 1963. Between the 1970s and 1990s, research in this field was focused on the mannose‐6‐phosphate receptor pathway and on the mechanisms underlying the sorting of lysosomal enzymes, on the identification of the molecular bases of LSDs, and on the development of tools and strategies to investigate lysosomal biology. The first attempts to treat these disorders by enzyme replacement therapy started in the 1990s. Current research is now focusing on the role of lysosomes as signaling platforms controlling cellular metabolism and on the development of new therapeutic approaches.

Knowledge on lysosomal storage diseases (LSDs) has been evolving for more than a century (Fig 1). The first phenotypes and clinical entities were described in the 19^th^ century (see Mehta *et al*, 2006 for review), long before the identification of lysosomes and the definition of their biochemistry and pathophysiology. At that time, the identification of these disorders was exclusively based on the characterization of clinical phenotypes and pathology.

The biochemical and cellular bases of LSDs were elucidated much later, when Christian de Duve’s work, corroborated by Alex B. Novikoff's electron microscopy observations, led to the identification of lysosomes as cellular catabolic stations (de Duve *et al*, 1955; Novikoff *et al*, 1956), and when the biochemical defects underlying some of the previously described clinical entities were discovered. Pompe disease was the first disorder to be identified as an LSD in 1963, when Henri G. Hers demonstrated that this disease is due to the lack of an acidic α‐glucosidase, similar to rat liver lysosomal maltase (Hers, 1963), and that this deficiency is responsible for glycogen storage in tissues. He also suggested that other diseases, such as the mucopolysaccharide storage diseases, might be due to enzyme deficiencies. Between 1960 and the mid‐70s, the biochemistry of LSDs was further characterized with the identification of the primary storage materials for other LSDs and the recognition of the respective enzyme deficiencies (Van Hoof, 1974).

For decades, the biology and function of lysosomes remained associated with their catabolic function, and LSD pathophysiology was seen as a direct consequence of defective degradation and disposal of complex substrates (Vellodi, 2005; Heard *et al*, 2010).

Between the late 1970s and 1990s, research in this field progressed with studies that considerably expanded the knowledge on lysosome biology and on the pathophysiology of LSDs. These studies led to the characterization of the mechanism underlying the sorting of lysosomal enzymes (Sly & Fisher, 1982) and to the identification of the molecular bases of clinical variability of LSDs (Beck, 2001; Kroos *et al*, 2012).

Following the recognition of the mannose‐6‐phosphate pathway's role in lysosomal enzyme trafficking and the availability of new technologies for the purification and manufacturing of lysosomal enzymes, the early 1990s inaugurated the first attempts to treat these disorders by replacing the defective enzyme activity (Barton *et al*, 1990; Barton *et al*, 1991).

At the same time, the introduction of novel technologies had a critical role in the study of LSDs. Techniques for targeted gene disruption and generation of knock‐out animal models for human LSDs provided tools to better characterize the pathophysiology of these disorders and to develop innovative therapeutic strategies (Pastores *et al*, 2013). Mass spectrometry technology allowed for lysosomal proteome analyses (e.g., mannose‐6‐phosphate glycoproteome) (Sleat *et al*, 2005) that led to the identification of lysosomal proteins and novel molecular bases of some LSDs.

In the past decade, a number of studies have expanded our knowledge of lysosomal biology and provided new and important insights on LSD pathophysiology. These studies identified the lysosomes as highly dynamic organelles involved in multiple cellular functions, including signaling, and able to adapt to environmental stimuli. (Settembre *et al*, 2013; Perera & Zoncu, 2016; Ballabio & Bonifacino, 2020).

Significant advancements have also facilitated greater understanding on the molecular and metabolic mechanisms underlying LSDs and the development of new therapeutic strategies for these diseases (Parenti *et al*, 2015; Platt *et al*, 2018; Ren & Wang, 2020). This review will focus on new tools and technologies to study LSDs, on emerging aspects of lysosomal biology, and on recent discoveries on the cellular and organismal consequences of lysosomal dysfunction.

## The biology of lysosomes, old concepts and new views

Lysosomes are membrane‐limited, ubiquitous, intracellular organelles involved in multiple cellular processes (Saftig & Klumperman, 2009; Ballabio & Bonifacino, 2020).

More than two hundred lysosomal‐resident proteins contribute to the biology and function of these organelles. Approximately 60 of them are acidic hydrolases (Lubke *et al*, 2009; Schroder *et al*, 2010). Most of them act as exoglycosidases or sulfatases and are localized to the lysosomal lumen. The others are localized at the lysosomal membrane and have multiple functions such as formation of a glycocalyx‐like layer, transport across the membrane, acidification, membrane stability, and mediating interaction between lysosomes and other cellular structures (Saftig & Klumperman, 2009; Ballabio & Bonifacino, 2020). In addition to lysosomal‐resident proteins, other proteins interact with the lysosome and participate to lysosomal function by being dynamically recruited to the lysosomal surface under certain conditions, for example, the transcription factor EB (TFEB), the mechanistic target of rapamycin complex 1 (mTORC1) (Perera & Zoncu, 2016; Ballabio & Bonifacino, 2020; Yim & Mizushima, 2020), the mTORC1 regulator tuberous sclerosis complex (TSC) (Dibble & Cantley, 2015), folliculin (FLCN) and FLCN‐interacting protein (FNIP) (Lawrence *et al*, 2019), the energy‐sensing complex AMP‐activated kinase (AMPK) (Zhang *et al*, 2014), and the signal transducer and activator of transcription‐3 (STAT3) (Liu *et al*, 2018).

The first function of normal lysosomes to be recognized is turnover of cellular constituents. Lysosomes are involved in the degradation of a broad variety of structurally diverse compounds, such as proteins, glycosaminoglycans, sphingolipids, oligosaccharides, glycogen, nucleic acids, and complex lipids. Cellular and extracellular materials and substrates destined for degradation reach lysosomes through different routes (endocytosis, phagocytosis, autophagy), or by direct transport. In this respect, lysosomes are part of a more complex pathway, referred to as the autophagy–lysosomal pathway (ALP). Autophagy plays a crucial role in cell homeostasis by controlling intracellular clearance and recycling of a variety of molecules and cellular components and also by sustaining cellular energy metabolism. Autophagy is a multistep pathway that involves autophagosome formation, cargo recruitment, and autophagosome–lysosome fusion. Importantly, autophagic function is entirely dependent on the ability of the lysosome to degrade and recycle autophagy substrates (Yim & Mizushima, 2020).

Much attention has been paid in recent years to the nutrient‐sensing function of lysosomes. Lysosomes are able to monitor the nutrient status of cells and to adjust their metabolism to changing energetic conditions. When nutrients are available mTORC1 is dynamically recruited to the lysosomal surface where it becomes active and promotes cellular anabolic processes (Saxton & Sabatini, 2017).

Recent studies have provided compelling evidence that lysosomal biogenesis and autophagy are controlled by the master transcriptional regulator transcription factor EB (TFEB) (Sardiello *et al*, 2009; Settembre *et al*, 2011). In addition to TFEB, another member of the MiT‐TFE family of transcription factors, TFE3, has a partially redundant function and is regulated in a similar manner (Martina *et al*, 2014; Raben & Puertollano, 2016).

TFEB subcellular localization and function is regulated by nutrient‐induced mTORC1‐mediated phosphorylation of specific serine residues (Settembre *et al*, 2012; Roczniak‐ Ferguson *et al*, 2012; Martina *et al*, 2012). On phosphorylation by mTORC1, TFEB is retained in the cytoplasm. A variety of stimuli leading to mTORC1 inactivation, such as starvation, induce TFEB dephosphorylation and nuclear translocation. Thus, the mTORC1‐TFEB regulatory axis enables lysosomes to adapt their function to environmental cues, such as nutrient availability (Ballabio & Bonifacino, 2020). Several additional conditions, such as infection, inflammation, physical exercise, endoplasmic reticulum stress, and mitochondrial damage, also promote TFEB nuclear translocation, highlighting the complexity of TFEB regulation (reviewed in Puertollano *et al*, 2018; Cinque *et al*, 2015).

Recent findings have added further complexity to the mechanisms by which mTORC1 phosphorylates TFEB (Napolitano *et al*, 2020). Unlike other substrates of mTORC1, TFEB is known to interact with RagGTPases (Martina & Puertollano, 2013). Due to this interaction, TFEB phosphorylation occurs through an mTORC1 substrate‐specific mechanism that is strictly dependent on the amino acid‐induced activation of RagC and RagD GTPases but is insensitive to Rheb activity induced by growth factors. This allows mTORC1 activity to be differentially regulated by different stimuli (Napolitano *et al*, 2020). This substrate‐specific regulation of TFEB by the mTORC1 pathway has a crucial role in Birt–Hogg–Dubé syndrome, a disorder characterized by benign skin tumors, lung, and kidney cysts and renal cell carcinoma (Kauffman *et al*, 2014; Calcagnì, *et al*, 2016) and caused by mutations in the lysosomal RagC/D activator folliculin (FLCN) (Napolitano *et al*, 2020).

Lysosomes are emerging as calcium (Ca^2+^) storage organelles. The concentration of free Ca^2+^ within the lysosome is around 500 µM, and therefore comparable to endoplasmic reticulum (ER) Ca^2+^ levels (Christensen *et al*, 2002). Ca^2+^ channels, such as transient receptor potential mucolipin‐1 (TRPML‐1, Mucolipin 1, MCOLN1) and the two‐pore channel (TPC), reside on the lysosomal membrane and have been shown to mediate local Ca^2+^ signals from intracellular compartments (e.g., mitochondria) (Xu *et al*, 2015; Xu & Ren, 2015). Lysosomal Ca^2+^ signaling participates in multiple cellular processes such as lysosomal acidification, the fusion of lysosomes with other cellular organelles, membrane trafficking and repair, autophagy, and formation of contact sites between the lysosome and the endoplasmic reticulum (Kilpatrick *et al*, 2013; Lloyd‐Evans & Waller‐Evans, 2019). Furthermore, lysosomal Ca^2+^ signaling is involved in the regulation of lysosomal biogenesis and autophagy through the activation of TFEB. Upon starvation, lysosomal Ca^2+^ release through TRPML1 activates the Ca^2+^‐dependent serine/threonine phosphatase calcineurin (CaN), which binds and dephosphorylates TFEB, thus promoting its nuclear translocation (Medina *et al*, 2015). TRPML1 also induces autophagic vesicle biogenesis through the generation of phosphatidylinositol 3‐phosphate (PI3P) and the recruitment of essential PI3P‐binding proteins to the nascent phagophore in a TFEB‐independent manner (Scotto‐Rosato *et al*, 2019).

## The nosography of LSDs

LSDs are multisystem disorders that are associated with a broad range of clinical manifestations affecting multiple organs and systems and causing visceral, ocular, hematologic, skeletal, and neurological signs. These manifestations are often highly debilitating, causing progressive physical and neurological disabilities.

In general, LSD presentations show broad variability (Beck, 2001), ranging from early‐onset (in some cases neonatal), severe clinical forms that often result in premature death of patients, to late‐onset, attenuated phenotypes that have a lesser impact on patient health and lifespan. Albeit individually rare, their cumulative incidence is estimated in approximately 1 in 5,000–7,500 births, with higher rates in specific populations. It is noteworthy that newborn screening programs for LSDs, now active in some countries, may in the future significantly change these estimates and will likely provide a more precise figure of LSD incidence (Spada *et al*, 2006; Hopkins *et al*, 2018; Wasserstein *et al*, 2019).

The nosography of LSDs has evolved over time, reflecting the advancements in the knowledge of lysosomal function and the cellular consequences of its dysfunction. The traditional classification based on the classes of stored substrates (glycosaminoglycans in the mucopolysaccharidoses, glycosphingolipids in the glycosphingolipidoses, glycoproteins in the oligosaccharidosis, etc) largely reflects the vision of lysosomes as catabolic organelles and is centered on the disease biochemistry. Accurate and exhaustive information on LSD classification and nosography can be found elsewhere (Platt *et al*, 2018).

With the improved knowledge on the molecular and cellular bases of LSDs, an alternative way of classifying LSDs has been proposed, based on the process that is defective in the biogenesis of lysosomal enzymes, rather than on the stored substrate (Platt, 2018). The majority of these disorders is due to deficiencies of soluble hydrolases that are involved in the sequential degradation of a specific substrate. Other disorders are due to deficiencies in upstream processes, such as post‐translational modifications (multiple sulfatase deficiency, MSD, due to the lack of an enzyme converting a cysteine into a formylglycine residue in the catalytic site of sulfatases) (Cosma *et al*, 2002; Dierks *et al*, 2002), or to defective sorting of lysosomal enzymes to lysosomes (mucolipidoses—ML—II and III, with deficient generation of mannose‐6‐phosphate) (Hickman & Neufeld, 1972). Others are due to mutations of non‐enzymatic activator proteins (saposin activator protein, SAP, deficiencies) (Tamargo *et al*, 2012), of solute carriers (cystinosis, infantile sialic acid storage disease) (Gahl *et al*, 1982; Verheijen *et al*, 1999) and other lysosomal membrane proteins (Danon disease, due to LAMP2 defective function) (Nishino *et al*, 2000; Tanaka *et al*, 2000), or are the consequence of defects in assembly and stability of multienzymatic complexes (galactosialidosis, due to cathepsin A deficiency) (d’Azzo *et al*, 1982).

## New technologies and cellular modeling to study lysosomal function in health and disease

**Figure 2:**
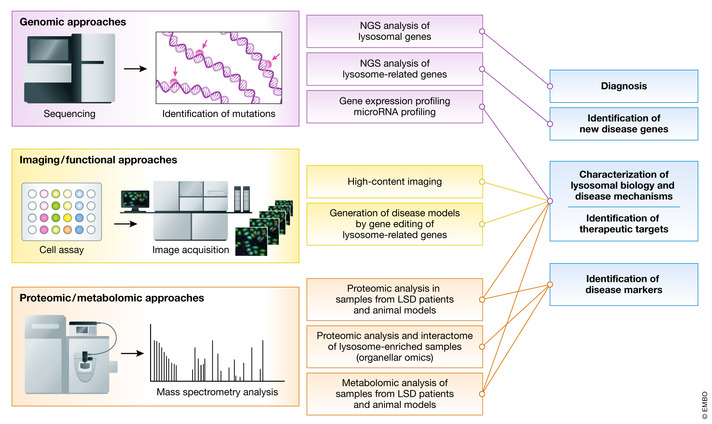
New technologies to study lysosomal function and biology New technologies, in most cases based on high‐throughput techniques combined with bioinformatic analysis, have been exploited for the diagnostic work‐up in patients with a suspected LSD, for the identification of new disease genes, for the search of disease biomarkers, for the characterization of lysosome biology and disease mechanisms, and for the identification and validation of new therapeutic targets.

Novel technologies have had significant impacts on the characterization of lysosome biology, the development of diagnostic tools for patients with a suspicion of LSD, and the identification and validation of new therapeutic targets (Fig 2) (Table 1). In several cases, these approaches are based on high‐throughput techniques combined with bioinformatic analysis of a large body of information (metabolomic, genomic, proteomic approaches). Novel approaches also include automated robotic‐based, miniaturized or cell‐based procedures (high‐content imaging) as well as innovative techniques that allow for manipulation of genetic information and generation of *in vitro* and *in vivo* models of disease.

**Table 1:** Examples of application of novel technologies for LSDs.

Technology	Applications	Examples of successful applications
Genomic sequencing	Diagnosis of LSD patients and identification of mutations of known genes Identification of mutations in genes not associated with LSD	Identification of new LSDs caused by mutation of the *VPS33A* (Pavlova *et al*, 2019) and *VPS16* (Steel *et al*, 2020) genes
Transcriptomic analysis	Information on pathways involved in disease pathophysiology Response to environmental conditions /pharmacological manipulations	Similarities between the microglia expression profiles of LSDs (mucolipidosis type IV mouse and Niemann‐Pick disease type C1) with common neurodegenerative disorders (Cougnoux *et al*, 2019)
Genome‐wide association studies	Identification of modifying factors Information on disease pathophysiology	Identification of a c.510C > T variant that may be predictive of clinical course and outcome in late‐onset Pompe disease patients (Bergsma *et al*, 2019)
microRNA sequencing	Identification of disease biomarkers that correlate with disease severity and assist in monitoring disease progression and efficacy of therapies Identification of pathways involved in disease pathophysiology	Identification of differentially expressed microRNAs potentially predictive of disease severity in Pompe disease (Tarallo *et al*, 2019)
Biochemical and metabolomic analyses	Support and validation of diagnosis Identification of disease biomarkers that correlate with disease severity, monitoring disease progression, monitoring efficacy of therapies Newborn screening Identification of pathways involved in disease pathophysiology	Identification of disease biomarkers for several LSDs (Boutin & Auray‐Blais, 2015; Reunert *et al*, 2015; Polo *et al*, 2019) Development of methods for simultaneous detection of multiple enzyme activities in dried blood spots suitable for newborn screening programs for several LSDs (Anderson, 2018; Donati *et al*, 2018; Kumar *et al*, 2019; Lukacs *et al*, 2019; Scott *et al*, 2020)
Cell‐based assays and high‐content imaging technologies	Identification of pathways involved in disease pathophysiology Screening for correctors and therapeutic agents	Development of multiplex staining assays that allow screening of FDA‐approved compounds and identification of correctors for cellular phenotypes of LSDs (Pipalia *et al*, 2006; Pugach *et al*, 2018)
Targeted gene knock‐out and genome editing—iPSc	Identification of pathways involved in disease pathophysiology Screening and validation of therapeutic agents Gene editing of mutant genes to correct disease‐causing mutations	CRISPR‐Cas9‐mediated generation of knock‐out models of LSDs, such as sphingolipidoses and Niemann‐Pick disease type C (Santos & Amaral, 2019)
Organellar omics	Information on lysosome biology Identification of pathways involved in disease pathophysiology Identification of disease biomarkers for correlations with disease severity, monitoring disease progression, monitoring efficacy of therapies	Identification of lysosomal proteome and interactome (Sleat *et al*, 2005; Abu‐Remaileh *et al*, 2017; Thelen *et al*, 2017; Rabanal‐Ruiz & Korolchuk, 2018)

### Genomic techniques and next‐generation sequencing (NGS)

A major advancement, particularly in the diagnostic approach to LSDs, was introduced by NGS, a powerful diagnostic tool based on technological platforms that allow sequencing of millions of small fragments of DNA in parallel. This technology has been used both through a targeted strategy with gene panels and by untargeted approaches based on whole‐exome sequencing.

Given the difficulties in the diagnostic work‐up, and due to overlapping clinical phenotypes in LSDs, patients with a clinical suspicion of these disorders are excellent candidates for the application of an NGS‐based diagnosis. Before the introduction of NGS, the traditional approach for the diagnosis of LSDs was based on a step‐by‐step, progressive process starting with physical examination, proceeding to metabolite identification in biological fluids, and leading to the exact diagnosis through the demonstration of an enzymatic deficiency (or the deficiency of a lysosomal function) and the identification of mutations in a specific gene (Winchester, 2014).

NGS‐based approaches are substantially changing this stepwise process. The molecular analysis and search for mutations in LSD‐related genes can be performed immediately after the clinical suspicion of LSD, whereas the functional analysis of the deficient enzyme (or a non‐enzymatic protein) offers a complementary approach to definitively confirm disease diagnoses. Such a diagnostic process may prove to be cost‐effective and by‐pass the need for multiple biochemical analyses or repeated hospital admissions. Some examples of this strategy are already available in the literature, with the development of a gene panel specific for 891 genes involved in the ALP function (Di Fruscio *et al*, 2015), or the identification of Pompe disease patients in cohorts of unidentified limb‐girdle muscular dystrophies (Savarese *et al*, 2018).

NGS‐based analysis also has the potential to identify new genes involved in lysosomal disorders, thus expanding the list of LSDs. Indeed, two novel disorders associated with lysosomal abnormalities and impaired vesicle trafficking were recently recognized through whole‐exome sequencing. Mucopolysaccharidosis plus (MPS‐plus), characterized by typical manifestations of mucopolysaccharidoses such as coarse facial features, skeletal abnormalities, hepatosplenomegaly, respiratory problems, and by remarkable levels of glycosaminoglycans excretion in urines (Kondo *et al*, 2017), was associated with mutations in the *VPS33A* gene (Pavlova *et al*, 2019). Loss‐of‐function *VPS16* gene mutations were found in patients with an early‐onset dystonia and with ultrastructural lysosomal abnormalities (Steel *et al*, 2020). Both *VPS33A* and *VPS16* genes encode for subunits of the homotypic fusion and vacuole protein sorting (HOPS) complex that is essential for lysosome fusion with endosomes and autophagosomes (Wartosch *et al*, 2015).

Genomic approaches may also contribute to the understanding of the complexity and clinical variability of LSDs. The application of these approaches to the study of LSDs is a vast and rapidly expanding field that has been comprehensively reviewed in recent years (Hassan *et al*, 2017; Davidson *et al*, 2018).

Genome‐wide association studies have been performed to identify modifiers in some LSDs, for example, Gaucher disease and Pompe disease. In Gaucher disease, single nucleotide polymorphisms (SNPs) within the *CLN8* gene locus were in linkage disequilibrium and associated with disease severity, possibly regulating sphingolipid sensing and/or in glycosphingolipid trafficking (Zhang *et al*, 2012). In Pompe disease, a c.510C > T variant was identified as a genetic modifier in late‐onset patients. This variant negatively influences pre‐mRNA splicing in patients carrying the c.‐32‐13T > G mutation, with significant correlations with residual alpha‐glucosidase activity and may be predictive of clinical course and outcome in late‐onset patients (Bergsma *et al*, 2019).

Transcriptomic analysis has been performed in several types of LSDs, such as Niemann‐Pick disease type C (Martin *et al*, 2019), mucopolysaccharidoses (Salvalaio *et al*, 2017; Peck *et al*, 2019), progranulin deficiency (Evers *et al*, 2017), Pompe disease (Turner *et al*, 2016), and Gaucher disease (Dasgupta *et al*, 2013), mucolipidosis type IV (Cougnoux *et al*, 2019). Although the animal models and the tissues differed in these analyses, it was possible to recognize a few common patterns in some diseases. In mucolipidosis type IV mouse microglia, the mixed neuroprotective/neurotoxic expression pattern showed similarities with that observed in Niemann‐Pick disease type C1 (Cougnoux *et al*, 2019). Of note, the changes observed in mucolipidosis type IV microglia overlapped with alterations found in common neurodegenerative disorders such as Alzheimer's, Parkinson's, and Huntington's diseases. The analysis of mid‐cervical cord in the mouse model of Pompe disease showed up‐regulation of pathways associated with cell death, proinflammatory signaling, and dysregulation of signal transduction pathways suggestive of impaired synaptic function and plasticity (Turner *et al*, 2016). Transcriptomic analysis in Gaucher disease mice revealed dysregulation of genes involved in cell growth and proliferation, cell cycle, heme metabolism, and mitochondrial dysfunction in liver, lung, and spleen (Dasgupta *et al*, 2013).

Albeit interesting, these data are largely affected by heterogeneity of the samples, indicating the need for integrated and systematic approaches in homogenous animal and cellular models.

In addition to disease mechanisms, NGS‐based analysis also has the potential to identify novel disease biomarkers. This approach has been used in recent studies that identified microRNAs as markers of Pompe disease, with correlations between disease phenotype and severity, and possibly with the response to enzyme replacement therapy (Cammarata *et al*, 2018; Tarallo *et al*, 2019; Carrasco‐Rozas *et al*, 2019). In Pompe disease, this analysis showed altered expression of microRNAs implicated in signaling pathways related to the pathophysiology of the disease like the mTOR and AMPK pathways, ubiquitin‐mediated proteolysis, cardiac hypertrophy, muscle atrophy, and regeneration, regulation of stem cells pluripotency and myogenesis. One of the differentially expressed microRNAs, miR‐133a, was identified as a potential marker of disease severity and response to therapy (Tarallo *et al*, 2019).

In Fabry disease, some of the dysregulated microRNAs are also related to the disease pathophysiology, such as miR‐199a‐5p and miR‐126‐3p that are known to be involved in endothelial dysfunction, and miR‐423‐5p and miR‐451a that are involved in myocardial remodeling (Cammarata *et al*, 2018).

### Biochemical, metabolomic analyses, and newborn screening for LSDs

New technologies for high‐throughput analysis of multiple metabolites have also significantly contributed to the diagnostics and monitoring of LSDs. The availability of measurable and objective disease markers remains a major challenge for many LSDs.

Clinical measures are often non‐specific (such as the 6‐min walk test, respiratory function tests, quality of life assessments) or can be influenced by inter‐ and intra‐investigator variance. Biochemical markers may complement these clinical measures and provide accessory, quantitative tools to follow disease course and patient response to therapies. The search for biochemical markers has benefited from a number of modern methodologies such as mass spectrometry or nuclear magnetic resonance‐based approaches. Mass spectrometry has become the most widely used platform for inborn metabolic diseases because of its ability to analyze a wide range of molecules in different body fluids, with optimal dynamic range and great sensitivity (Costanzo *et al*, 2017). Metabolome analyses have been performed in some LSDs, such as some mucopolysaccharidoses (Fu *et al*, 2017; Tebani *et al*, 2019), Fabry disease (Boutin & Auray‐Blais, 2015), Pompe disease (Sato *et al*, 2017), Niemann‐Pick disease type C (Fan *et al*, 2013; Maekawa *et al*, 2015; Probert *et al*, 2017), neuronal ceroid lipofuscinoses (Sindelar *et al*, 2018), or in some sphingolipidoses (Polo *et al*, 2019).

This approach has allowed the identification of markers that may serve to facilitate early‐stage diagnosis and monitoring of response to therapy, for example, galabiosyl ceramide analogs in Fabry disease (Boutin & Auray‐Blais, 2015), oxysterols in Niemann‐Pick disease type C2 (Reunert *et al*, 2015), and various sphingolipids in some sphingolipidoses (Reunert *et al*, 2015). However, some issues need to be addressed in these studies, such as those related to the heterogeneity of samples used for the analyses and the number of patients required to be recruited in order to satisfy statistical significance (Percival *et al*, 2020).

Tandem mass spectrometry and digital microfluidic fluorimetry (DMF‐F) have found important applications in the context of neonatal screening programs (Burlina *et al*, 2018; Gelb *et al*, 2019).

The first trials of newborn screening for LSDs started about two decades ago with an immunoassay for the lysosomal marker lysosomal‐associated membrane protein‐1 (LAMP‐1) in dried blood spots (Ranierri *et al*, 1999). Other approaches followed, such as a multiplex immune‐quantification assay of lysosomal enzymes (Meikle *et al*, 2006), enzyme assays by DMF‐F, and tandem mass spectrometry (Gelb *et al*, 2019). Methods to test lysosomal enzyme activities in dried blood spots suitable for newborn screening programs have been developed for Fabry disease, Gaucher disease, Krabbe disease, Niemann‐Pick A/B, Pompe disease, and mucopolysaccharidoses, and for less prevalent disorders such as alpha‐mannosidosis, alpha‐fucosidosis, lysosomal acid lipase deficiency, and ceroid lipofuscinosis 1 and ceroid lipofuscinosis 2 (Anderson, 2018; Donati *et al*, 2018; Kumar *et al*, 2019; Lukacs *et al*, 2019; Scott *et al*, 2020). In most instances, these methods allow for simultaneous detection of multiple LSDs (Kumar *et al*, 2019; Lukacs *et al*, 2019; Hong *et al*, 2020).

These screening programs, already active in several countries (Schielen *et al*, 2017), have had a significant impact on the care of LSDs, allowing early diagnosis and timely access to therapies (Chien *et al*, 2009) and have changed the figure of LSD prevalence in different countries and populations (Spada *et al*, 2006).

### Cell‐based assays and high‐content imaging technologies

Together with robotics, the development of cell‐based assays in combination with high‐content imaging technologies for screening has represented a major and fascinating advance for diseases that are associated with an evident cellular phenotype. Cell‐based high‐content imaging usually exploits automated microscopy and complex image algorithms to extract multidimensional information from hundreds of samples, such as cell morphology, fluorescence intensity, or distribution of fluorescent markers within cells (Bellomo *et al*, 2017). Results usually have very high statistical power, due to the high number of cells that can be analyzed allowing averaging of large amount of data. The major advantage, however, is represented by the possibility of multiplexing different assays simultaneously in integrated cell populations, cell subpopulations, individual cells, and subcellular structures within a given population (Zanella *et al*, 2010; Peravali *et al*, 2011). For these reasons, high‐content imaging is currently used to study disease mechanisms by loss‐of‐function and gain‐of‐function studies, as well as in preclinical drug discovery including target identification, lead optimization, assay validation, or primary and secondary screenings (Bellomo *et al*, 2017).

Several studies substantiate the potential of cell‐based screening approaches to identify candidate molecules for the treatment of LSDs, some of them exploiting high‐content imaging assays. For example, lysotracker and filipin fluorescence staining assays, or multiplex staining with dual markers filipin and anti‐LAMP1 have been exploited to screen FDA‐approved compounds and identify correctors of Niemann‐Pick disease type C1 (Pipalia et a, 2006; Pugach *et al*, 2018). One such drug is the antimicrobial alexidine dihydrochloride that appeared to promote increases in *NPC1* transcript and mature protein and to be a potent cholesterol‐reducing drug. A cell‐based assay was also developed to detect arylsulfatase A residual activity in cells from patients with metachromatic leukodystrophy (Geng *et al*, 2011). Furthermore, a cell‐based assay was used to identify three compounds that enhance galactocerebrosidase activity (Jang *et al*, 2016). Another phenotypic‐based approach was used to identify modulators of autophagy in a murine neuronal cell model of CLN3 disease and led to the identification of compounds that normalized lysosomal positioning and promote clearance of storage material (Petcherski *et al*, 2019).

In summary, together these data, albeit preliminarly, suggest that cell‐based screening approaches may lead to the development of novel therapeutics for lysosomal storage diseases.

### Targeted gene knock‐out and genome editing techniques

Recently, the development of CRISPR/Cas9 approaches for genome editing and the technology for the generation of induced pluripotent stem cells (iPSCs) from skin or blood samples have opened a new era for the development of disease‐relevant cellular models of genetic diseases. These tools have found particular application for the generation of drug‐based screening systems and for the study of disease pathophysiology. iPSCs derived from patients are pluripotent and capable of differentiating into virtually any cell type, including disease‐relevant neuronal subtypes (Khurana *et al*, 2015) that display major common features of LSD pathology such as autophagic, lysosomal maturation, and mitochondrial defects (Lojewski *et al*, 2014). However, a major inconvenience of patient‐derived iPSCs is their genetic diversity. The use of (CRISPR)‐Cas9 gene editing to introduce specific genetic changes into a parental pluripotent line allows the production of isogenic lines representing selected mutations of LSD genes that can more readily be compared (Chaterji *et al*, 2017). CRISPR‐Cas9 is currently being exploited to generate knock‐out models for the study of LSDs. An exhaustive review about modeling of both cellular and *in vivo* modeling of rare sphingolipidoses and Niemann‐Pick disease type C, using this technology, has been published recently (Santos & Amaral, 2019).

Both CRISPR‐Cas9‐ and iPSC‐based approaches offer unprecedented opportunities for the generation of cellular models of LSDs. These can be used for the identification of new pathways involved in the physiology and pathogenesis of LSDs and for drug screenings to identify of new drugs for the therapy of these diseases. Additionally, CRISPR/Cas9 can be by itself an alternative therapeutic option for treating LSDs by correcting disease‐causing mutations, both *in vitro* and *in vivo* (Schwank *et al*, 2013; Wu *et al*, 2013; Xie *et al*, 2014).

Targeted gene knock‐out and knock‐in technologies have found important applications in the generation of *in vivo* models of LSDs. Compared to *in vitro* systems, animal models have major advantages as they provide opportunities to study pharmacokinetics, bioavailability, toxicity of therapeutic agents, and to evaluate critical endpoints such as metabolic responses in organs and tissues, and functional measures (Moro & Hanna‐Rose, 2020). Animal models have been developed for nearly all LSDs (Vaquer *et al*, 2013), the majority in small, prolific species such as mice and rats (Vaquer *et al*, 2013; Gurda & Vite, 2019). Knock‐in animal models have peculiar advantages as they allow characterization of the phenotypic, pathologic, and functional consequences of specific mutations found in patients (Praggastis *et al*, 2015) or for testing the response of such mutations to experimental treatments (Khanna *et al*, 2010).

### Organellar omics

The characterization of the lysosomal proteome has been exploited as an important tool for the understanding of lysosomal dysfunction in human disease and of LSD pathophysiology. Proteomic analysis of lysosomes is mainly based on tandem mass spectrometry and has already proven to be a suitable strategy for the analysis of proteins interactions (Sleat *et al*, 2005), for the identification of biomarkers (Cologna *et al*, 2012; Matafora *et al*, 2015), for the identification of the molecular bases of LSDs, such as CLN2 (Sleat *et al*, 1997) and NPC2 (Naureckiene *et al*, 2000), and for the identification of novel potential lysosomal membrane transporters (Chapel *et al*, 2013).

Proteome analyses in LSD animal models and in selected samples from patients have been performed for Krabbe disease (Pellegrini *et al*, 2019), mucolipidosis III (Di Lorenzo *et al*, 2018) ceroid lipofuscinoses (Sleat *et al*, 2019), Niemann‐Pick type C1 (Pergande *et al*, 2019), in some mucopolysaccharidoses (Yuan *et al*, 2019), and others. In recent years, in light of the emerging role of lysosomes as signaling platforms controlling cellular metabolism, this approach has attracted renewed and growing interest. Depending on the studies, approximately 200 proteins have been identified, including *bona fide* lysosomal and lysosomal‐associated proteins. Interestingly, most of the lysosomal‐associated proteins correspond to members of the mTORC1 complex (Thelen *et al*, 2017; Rabanal‐Ruiz & Korolchuk, 2018). Searching for lysosomal terms in gene ontology and protein databases resulted in the identification of at least 500 proteins (Mi *et al*, 2019; The UniProt Consortium, 2019).

However, this approach is associated with important challenges related to the limited sensitivity of technologies in detecting low abundance proteins in lysosomes, lack of spatial information about the localization of the identified proteins in whole‐cell analysis, and interaction with other organelles. In recent years, methods to reduce the biomolecular complexity of a sample by isolation and purification of individual cellular organelles have been developed (Diettrich *et al*, 1998; Chen *et al*, 2005; Walker & Lloyd‐Evans, 2015; Tharkeshwar *et al*, 2017). These methods include density gradient centrifugation of cellular or tissue homogenates, use of magnetic iron oxide (FeO)‐coated high‐molecular‐weight dextran particles to purify lysosomes from mammalian cells (Diettrich *et al*, 1998; Chen *et al*, 2005), and delivery of superparamagnetic iron oxide nanoparticles (SPIONs) to lysosomes by endocytosis (Walker & Lloyd‐Evans, 2015). In this regard, it is important to mention the importance of the protein localization database based on careful subcellular localization studies combined with MS analysis (Prolocate, http://prolocate.cabm.rutgers.edu). More recent approaches are based on immunoaffinity enrichment of lysosomes from cells expressing a lysosomal transmembrane protein (i.e., TMEM192) fused to three tandem human influenza virus hemagglutinin (HA) epitopes using an antibody against HA conjugated to magnetic beads (Abu‐Remaileh *et al*, 2017; Wyant *et al*, 2018). This method is rapid at extracting highly pure lysosomes and may represent an important advance in the analysis of the lysosomal proteome and in the quantitative profiling of metabolites derived from the action of lysosomal enzymes or from the activity of lysosomal transporters under various cell states (Abu‐Remaileh *et al*, 2017).

Changes in the amounts of specific metabolites within the lysosome might uncover novel aspects involved in the pathophysiology of complex LSD and lead to the identification of aberrant accumulation of specific cargo or metabolites that could be used as biomarkers to test the efficacy of novel therapies.

## How defects of lysosomal functions lead to disease

Historically, the pathology and the clinical manifestations of lysosomal disorders have been considered as direct consequences of the storage of inert substrates in tissues.

Indeed, manifestations such as visceromegaly, skin thickness, skeletal dysmorphisms, and ocular signs (corneal opacities, cherry‐red spot) might easily be viewed as the results of excessive undegraded substrates in cells and extracellular matrix.

**Figure 3:**
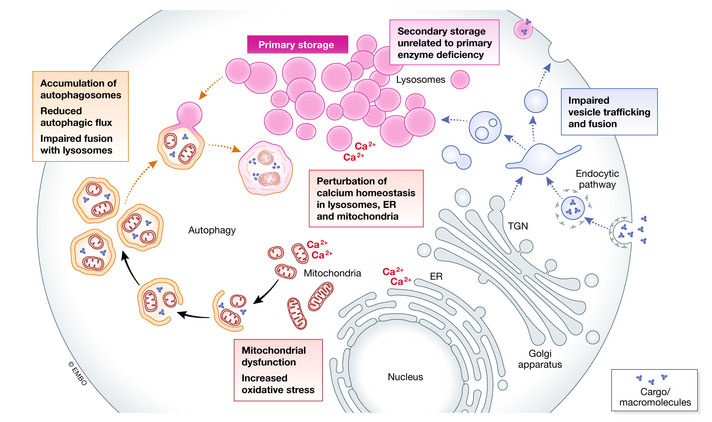
The mechanisms involved in the pathophysiology of LSD The accumulation of undegraded substrates triggers multiple events that are currently emerging as important players in the pathogenesis of LSDs. These events include storage of secondary substrates unrelated to the defective enzyme; abnormal composition of membranes and aberrant fusion and intracellular trafficking of vesicles; impairment of autophagy; perturbation of calcium homeostasis; and mitochondrial dysfunction and oxidative stress. In addition to the events shown in the figure, in several LSDs storage triggers dysregulation of signaling pathways and activation of inflammation.

This concept has been questioned by the new vision of lysosomal functions. Given the central role of lysosomes in cellular homeostasis and metabolism, it has been speculated that storage is just the “instigator” of a number of secondary events (Clarke, 2011) and that accumulation of undegraded substrates is able to prime complex pathogenetic cascades that are in fact responsible for LSD manifestations. Multiple and diverse events are now emerging as players in the pathogenesis of LSDs. Specifically, these events include storage of secondary substrates unrelated to the defective enzyme, abnormal composition of membranes and aberrant fusion and intracellular trafficking of vesicles, altered autophagic flux and accumulation of autophagic substrates, dysregulation of signaling pathways and activation of inflammation, abnormalities of calcium homeostasis, mitochondrial dysfunction, and oxidative stress (Ballabio & Gieselmann, 2009; Platt *et al*, 2018) (Fig 3).

### Secondary storage

Secondary storage of unrelated and heterogeneous substrates has been extensively documented in several LSDs. For example, in mucopolysaccharidoses types I, II, IIIA, VI, and VII, characterized by primary storage of glycosaminoglycans, biochemical analysis of brain has shown that gangliosides GM2 and GM3 are also consistently and substantially elevated (Walkley & Vanier, 2009). Accumulation of GM2 and GM3 gangliosides has also been documented in a variety of other LSDs, including Niemann‐Pick type A and type C1 (Zervas, *et al*, 2001), mucolipidosis type IV (Micsenyi *et al*, 2009) neuronal ceroid lipofuscinoses (Jabs *et al*, 2008), and alpha‐mannosidosis (Goodman *et al*, 1991).

Secondary storage is thought to have a substantial role in the pathophysiology of LSDs. This idea is supported by the finding that secondary substrates localize in the brain areas that are most affected by the disease pathology (Tobias *et al*, 2019; Viana, *et al*, 2020) and that depletion of secondary substrates in experimental conditions (e.g., GM3 in the Niemann‐Pick type C1 mouse model) results in amelioration of neuropathology and disease manifestations (Lee *et al*, 2014). Secondary storage may also impair vesicle trafficking. Cholesterol and other lipids, for example, have been shown to impair the endo‐lysosomal system (Sobo *et al*, 2007; Walkley & Vanier, 2009), and the function of soluble N‐ethylmaleimide‐sensitive factor attachment protein receptors (SNAREs) (Fraldi *et al*, 2010; Sambri *et al*, 2017), which are critical for the fusion of cellular membranes (Lang *et al*, 2001).

These effects are not only limited to lysosomes but also implicated in the accumulation in other compartments of toxic storage materials, including multiple aggregate‐prone proteins, α‐synuclein, prion protein, Tau, amyloid β, and damaged mitochondria (Fraldi *et al*, 2016) that are known to be associated with common neurodegenerative disorders, such as Alzheimer's, Parkinson's, and Huntington's disease. Alpha‐synuclein accumulation has been found in several LSDs (Settembre *et al*, 2008a; Shachar *et al*, 2011; Di Malta *et al*, 2012) and suggests a link between alpha‐synuclein aggregation toxicity and neurodegeneration in LSDs. Extensive work has been done about beta‐glucocerebrosidase deficiency as a risk factor for the development of Parkinsonism (Aharon‐Peretz *et al*, 2004; Gan‐Or *et al*, 2008; Blanz & Saftig, 2016).

### Impairment of autophagy

As lysosomes are the terminal compartment of the ALP, a general impairment of this pathway and of its critical functions in cell homeostasis is an obvious and expected finding in LSDs. A block of autophagy was initially recognized in a few of these disorders, namely in mucopolysaccharidosis type IIIA and in MSD (Settembre *et al*, 2008a), as well as in Pompe disease in which the accumulation of large pools of autophagic debris is a typical and consistent feature of skeletal muscle pathology (Fukuda *et al*, 2006a; Shea & Raben, 2009) and appears to be associated with dysregulation of mTORC1 and AMPK signaling (Lim *et al*, 2017).

Autophagic vesicle accumulation, together with increased poly‐ubiquitinated proteins and dysfunctional mitochondria, has now been reported also in several other LSDs, such as some mucopolysaccharidoses, sphingolipidoses (Gaucher and Fabry disease, Niemann‐Pick type C), mucolipidoses (type II, III and IV), Danon disease, and some neuronal ceroid lipofuscinoses (CLN 3, 10) (Liebermann *et al*, 2012).

The impairment of autophagy has important and deleterious consequences and is thought to contribute substantially to LSD pathophysiology. In Pompe disease, for example, the presence of autophagic accumulation was shown to impair the contractile function of muscles (Drost *et al*, 2005) and to affect the trafficking of recombinant enzymes used for enzyme replacement therapy (Fukuda *et al*, 2006b).

In neurons, a dysfunction of the ALP is thought to be involved in the pathophysiology of neurodegeneration (Hara *et al*, 2006; Komatsu *et al*, 2006; Monaco *et al*, 2020). The impairment of the ALP has also been shown to affect extracellular matrix formation and skeletal development and growth in chondrocytes from the mouse model of MPS VII (Bartolomeo *et al*, 2017; Settembre *et al*, 2018).

### Mitochondrial dysfunction

One of the primary functions of autophagy is to execute mitochondrial turnover (Plotegher & Duchen, 2017; Wang & Wang, 2019). Thus, it is not surprising that mitochondrial dysfunction is emerging as an important player in the pathophysiology of LSDs. Substantial evidence supports the existence of a crosstalk and reciprocal functional relationships between mitochondria and lysosomes (Pshezhetsky, 2015). While defective autophagy affects mitochondrial quality control pathways and causes accumulation of damaged mitochondria, in turn mitochondrial dysfunction can impair lysosomal functions, such as acidification by the acidic pump V‐ATPase that relies on the ATP generated by mitochondria (Stepien *et al*, 2020).

Perturbations in mitochondrial function and homeostasis have been recognized in several LSDs, including sphingolipidoses (Gaucher disease, Niemann‐Pick disease type C, Krabbe disease), gangliosidoses, some mucopolysaccharidoses, multiple sulfatase deficiency, and neuronal ceroid lipofuscinoses (Plotegher & Duchen, 2017; Stepien *et al*, 2020) and have been proposed as one of the mechanisms underlying neurodegeneration (Martins *et al*, 2015; Saffari *et al*, 2017; Annunziata *et al*, 2018).

Multiple mitochondrial defects were found in the mouse model of Pompe disease, including mitochondrial calcium excess, increased reactive oxygen species, decreased mitochondrial membrane potential, and decreased oxygen consumption and ATP production (Lim *et al*, 2015). Increased oxidative stress, elevation of reactive oxygen species (ROS), and enhanced susceptibility of cells to mitochondria‐mediated apoptotic insults are obvious consequences of defects in mitophagy and mitochondrial dysfunction (Filomeni *et al*, 2015). Oxidative stress was observed in animal models of mucopolysaccharidoses type IIIB (Villani *et al*, 2009), type I (Donida *et al*, 2015), and type IIIA (Arfi *et al*, 2011) and in blood samples from patients affected by mucopolysaccharidoses types I (Pereira *et al*, 2008) and type II (Filippon *et al*, 2011).

### Alteration of signaling pathways and inflammation

Non‐physiologic activation of signal transduction pathways by storage compounds is another consequence of storage in LSDs. Stored materials may interfere with normal ligand–receptor interactions, modify receptor responses, influence internalization and recycling of receptors, and lead to altered activation of signaling pathways involved in cellular transport and vesicle trafficking, calcium homeostasis, oxidative stress, morphogen signaling, inflammatory and innate immune responses, and cell death (von Zastrow & Sorkin, 2007; Ballabio & Gieselman, 2009; Fiorenza *et al*, 2018; Platt *et al*, 2018).

Neuroinflammation and bone involvement are paradigm examples of manifestations related to altered signaling. Neuroinflammation has been reported in a large variety of LSDs, such as mucopolysaccharidoses (Zalfa *et al*, 2016; Viana *et al*, 2020; Heon‐Roberts *et al*, 2020), sphingolipidoses (Potter & Petryniak, 2016; Fiorenza *et al*, 2018; Cougnoux *et al*, 2019), neuronal ceroid lipofuscinoses (Groh *et al*, 2016), and gangliosidoses (Utz *et al*, 2015). In some mucopolysaccharidoses, it has been shown that structurally anomalous glycosaminoglycans may mimic lipopolysaccharide, an endotoxin of gram‐negative bacteria, and activate the Toll‐like receptor 4 (TLR4) innate immune responses. As a consequence of TLR4 activation, secretion of proinflammatory cytokines increases together with the activation of tumor necrosis factor (TNF)‐alpha (Simonaro *et al*, 2010; Parker & Bigger, 2019). Furthermore, the activation of an atypical pattern of interferon downstream signaling, involving both interferon (IFN)‐gamma‐ and IFN‐alpha‐responsive genes, was detected in the cerebellum of the Niemann‐Pick disease type C1 mouse model, resulting in the elevation of IFN‐gamma‐responsive cytokines (Shin *et al*, 2019).

Interestingly, aberrant morphogen signaling has emerged as a possible mechanism that may explain some clinical features of LSDs, such as skeletal dysmorphisms, traditionally viewed as a direct consequence of substrate accumulation (Fiorenza *et al*, 2018). For example, excess of accumulated glycosaminoglycans and defective proteoglycan desulfation have been shown to alter fibroblast growth factors‐2 (FGF2)‐heparan sulfate interactions and the FGF2 signaling pathway in a murine model of MSD (Settembre *et al*, 2008b), and bone morphogenetic protein (BMP)‐4 signaling activity in mucopolysaccharidosis type I cells (Pan *et al*, 2005).

Altered FGF2 and Indian hedgehog distribution and impaired FGF2 signaling have been observed in growth plates from mucopolysaccharidosis type I mice (Kingma *et al*, 2016). In a zebrafish model of mucopolysaccharidosis type II, perturbations of glycosaminoglycan catabolism were associated with aberrant distribution and signaling of morphogens, such as sonic hedgehog (Shh), dysregulation of the Shh and Wnt/β‐catenin signaling, and aberrant heart development and atrioventricular valve formation (Costa *et al*, 2017).

Shh dysregulation, with severely disturbed subcellular localization of the Shh effectors Patched (Ptch) and Smoothened (Smo), and of ciliary proteins were found in Niemann‐Pick disease type C1 mice. Dysregulation of Shh signaling has been associated with shortening of primary cilium length and reduction in ciliated cells in animal brains and was proposed as a mechanism underlying abnormal cerebellum morphogenesis in this mouse model (Canterini *et al*, 2017; Formichi *et al*, 2018).

Abnormalities of Ca^2+^ signaling are thought to play an important role in LSDs (Lloyd‐Evans & Waller‐Evans, 2019; Liu & Lieberman, 2019). The importance of this signaling pathway for LSD pathophysiology was revealed by mucolipidosis type IV. This disorder is due to mutations in the mucolipin 1 (*MCOLN1)* gene that encodes the lysosomal Ca^2+^‐releasing channel TRPML1. Mucolipidosis IV pathology is the consequence of defective TRPML1 function and aberrant Ca^2+^ signaling, and is characterized by impaired vesicle trafficking and extensive storage of granular material and lamellar and concentric bodies (LaPlante *et al*, 2004). Ca^2+^ abnormalities have also been found in other LSDs and thought to participate in disease pathophysiology. In Niemann‐Pick type C1 mutant cells, for example, remarkable reduction in the acidic compartment calcium stores was observed, compared to wild‐type cells, likely due to sphingosine storage that induces calcium depletion in lysosomes, possibly through an inhibitory effect on Na^+^/Ca^2+^ exchangers (Lloyd‐Evans *et al*, 2008; Lloyd‐Evans & Platt, 2011).

## Current and future therapies

### Enzyme replacement therapy

Given that the majority of LSDs are due to the deficiency of a lysosomal hydrolase, the primary approach, and the first to be explored, was based on replacing the defective activity with a wild‐type functional enzyme. This strategy was pursued through hematopoietic stem cell transplantation and enzyme replacement therapy (ERT), which are based on the concept that lysosomal enzymes can be taken up by cells and correctly delivered to lysosomes through the mannose‐6‐phosphate pathway.

After the pioneering studies in Gaucher disease (Barton *et al*, 1990; Barton *et al*, 1991) that demonstrated the feasibility and the efficacy of ERT in the non‐neuronopathic forms of this disorder, this approach is currently considered the standard treatment for several LSDs, including the most prevalent of these disorders, such as Gaucher disease, Fabry disease, Pompe disease, some mucopolysaccharidoses, and a few ultra‐rare disorders (e.g., lysosomal acid lipase deficiency, mucopolysaccharidosis type VII) and is in clinical development for others (Platt *et al*, 2018; Poswar *et al*, 2019). However, despite remarkable success in treating some aspects of LSDs, we have learned from the experience of many years that ERT has limitations. Recombinant enzymes are immunogenic and may induce immune responses, particularly in cross‐reactive immunologic material (CRIM)‐negative patients (Berrier *et al*, 2015). Development of antibodies against the therapeutic enzyme may impact on the efficacy of therapies or cause immune‐mediated severe adverse reactions that require laborious and expensive protocols for induction of immune tolerance (Desai *et al*, 2019). ERT is based on periodic infusions, often requiring the use of intra‐venous devices that are associated with risk of life‐threatening infections. Thus, the impact on the quality of life of patients and their caregivers is substantial (Wyatt *et al*, 2012; Platt, 2018). In addition, costs of therapies are high and represent an economical burden for healthcare systems (Wyatt *et al*, 2012).

An additional and major issue is the insufficient biodistribution of the recombinant enzymes used for ERT, leading to inability to reach therapeutic concentrations in specific tissues. Recombinant enzymes are large molecules, often unable to cross anatomical and functional barriers. In several LSDs, the main target tissues, where the correction of enzyme activity is required to clear storage and improve pathology, are in fact the most difficult to reach (van Gelder *et al*, 2012). For these reasons, second‐generation recombinant enzymes with improved targeting properties or pharmacodynamics are currently under development, for example, avalglucosidase‐apha, a glycoengineered GAA, for the treatment of Pompe disease (Pena *et al*, 2019; Xu *et al*, 2019) and pegunigalsidase alfa, a PEGylated covalently cross‐linked alpha‐galactosidase A, for the treatment of Fabry disease (Schiffmann *et al*, 2019).

The inability of recombinant enzymes to cross the blood–brain barrier has major clinical relevance, as the majority of LSDs can be associated with neurological manifestations. Intrathecal or intraventricular delivery of enzyme has been proposed for some mucopolysaccharidoses (Giugliani *et al*, 2018), metachromatic leukodystrophy (I Dali *et al*, 2020), and is now an approved treatment for neuronal ceroid lipofuscinosis 2, in which intraventricular infusions of cerliponase‐alpha reduced disease progression (Schulz *et al*, 2018). The use of chimeric lysosomal enzymes, in which the therapeutic enzyme is conjugated with different peptides or antibody components that exploit interactions with specific receptors and mediate transport across the blood–brain barrier, is also a strategy that is currently being investigated (Sonoda *et al*, 2018; Pardridge *et al*, 2018; Yogalingam *et al*, 2019).

### Pharmacological therapy

Indeed, LSDs have proven to be an extraordinary field of investigation with the development of multiple and diverse therapeutic strategies that hit different targets in the pathogenetic cascade of these disorders (Cox, 2015).

One of these strategies was aimed to restore the equilibrium of the so‐called “storage equation” (i.e., the balance between the amount of substrate that is delivered to lysosomes for degradation, and the amount of enzymes that are involved in its breakdown) by reducing flux of substrates to lysosomes with small‐molecule inhibitors of substrate synthesis (substrate reduction therapy—SRT). The first of such compounds to be used was the imino sugar Miglustat (N‐butyl‐deoxynojirimicin), a reversible inhibitor of glucosylceramide synthase, that catalyzes the formation of glucocerebroside and thereby initiates the glycosphingolipid biosynthetic pathway (Pastores & Barnett, 2003). Miglustat showed efficacy in correcting some clinical and biochemical parameters in Gaucher disease patients (Cox, 2005; Charrow & Scott, 2015). For its effect on this early step of the glycosphingolipid biosynthetic pathway, Miglustat has also been proposed for the treatment of GM1 and GM2 gangliosidoses, with a slowed disease progression (Poswar *et al*, 2019; Fischetto *et al*, 2020), and is approved for the treatment Niemann‐Pick disease type, in which gangliosides GM2, GM3 gangliosides, and other glycosphingolipids play a role in the pathogenesis of neurological manifestations (Zervas *et al*, 2001). Other substrate‐reducing molecules are now in clinical development or approved for the treatment of Gaucher and Fabry disease, such as eliglustat tartrate, venglustat, and lucerastat (Felis *et al*, 2019; Poswar *et al*, 2019). This approach is attractive, as substrate‐reducing drugs are small molecules that reach therapeutic concentrations in tissues, including those that are difficult to reach by ERT, and can be taken orally, with minimal impact on patient quality of life.

Newer approaches, based on different strategies, are now in a phase of advanced development. In most cases, they are also aimed at increasing or replacing the defective enzyme activity. Pharmacological chaperone therapy (PCT) is based on the use of small‐molecule drugs that enhance the stability of mutant enzyme proteins with residual activity, through specific non‐covalent interactions with the target enzymes, thus favoring their trafficking to lysosomes (Parenti *et al*, 2015).

PCT is now approved for the treatment of patients affected by Fabry disease. Migalastat (1‐deoxygalactonojirimycin), an active site‐directed imino sugar reversible inhibitor of alpha‐galactosidase A, was shown to paradoxically rescue the activity and the stability of this enzyme in cells from Fabry disease patients, opening the way to further development of this approach (Fan *et al*, 1999). This compound has shown clinical efficacy on different clinical manifestations of Fabry disease (Germain *et al*, 2016; Hughes *et al*, 2017; Lenders *et al*, 2020) and is now approved for the treatment of patients with amenable *GLA* gene mutations. PCT has evident advantages compared with ERT, as chaperones are small‐molecule drugs that can be taken orally by patients and are expected to penetrate across membranes and physiological barriers, thus reaching therapeutic concentrations in multiple tissues. On the other hand, a major limitation of this approach is the possibility to treat only patients carrying specific mutations (Benjamin *et al*, 2017). Concerns on the use of PCT have also been raised as chaperones interact with the catalytic sites and are thus reversible competitive inhibitors of their target enzymes. For the treatment of Fabry disease, this risk has been eluded using a protocol based on discontinuous, every other day administration (Germain *et al*, 2016), taking advantage of the short half‐life of the drug compared with that of alpha‐galactosidase A. New, allosteric drugs that interact with non‐catalytic domains of the enzyme and are thus non‐inhibitory may represent an alternative strategy to minimize the risk of unwanted enzyme inhibition (Porto *et al*, 2012; Parenti *et al*, 2015a).

The use of proteostasis modulators has also been proposed to rescue mutant, unstable lysosomal enzymes (Mu *et al*, 2008; Fog *et al*, 2018; Seemann *et al*, 2020). However, the drugs used for this approach are non‐specific and may be associated with significant adverse effects.

The demonstration of a synergy between PCT and ERT attracted further interest on this approach. This effect was first demonstrated *in vitro* and *in vivo* in cells from patients with Pompe disease and in the murine model of this disease (Porto *et al*, 2009; Khanna *et al*, 2012). This synergy was translated into clinical trials (Parenti *et al*, 2014; Kishnani *et al*, 2017) and is now under further clinical development (Data ref: ClinicalTrials.gov NCT04138277). The advantage of this synergy, compared with the “traditional” use of chaperones, is that the effect of the drug is directed toward the recombinant enzyme used for ERT, and not to the endogenous mutant enzyme. Thus, the effect of chaperones with this approach is mutation‐independent and can in principle be extended to all patients on ERT. Also, the administration of the chaperone is limited to the time of the ERT infusion (e.g., every other week in Pompe disease), with less risk of undesired effects of the drug.

### Gene therapy

Gene therapy holds great promise and is under advanced clinical development for several LSDs. The approaches used so far for gene therapy of LSDs are based both on the use of adeno‐associated viral (AAV) vectors *in vivo* and of lentiviral vectors *ex vivo*. AAV‐mediated *in vivo* gene transfer is based on injection of a vector carrying the transgene under the control of ubiquitous or organ‐specific promoters. *Ex vivo* gene therapy is based on the correction of patient's cells, such as hematopoietic stem cells, followed by genetic modification *in vitro* and re‐implantation in the patients of the modified cells. Both approaches imply both local correction of a target tissue/organ and cross‐correction of distant tissues/organs by secreted enzymes that are internalized through the mannose‐6‐phosphate receptor pathway (Sands & Davidson, 2006).

Quite encouraging results have been obtained using the *ex vivo* approach in metachromatic leukodystrophy (Biffi *et al*, 2013; Sessa *et al*, 2016). Patients treated early showed improved course or complete prevention of disease manifestations, associated with improved brain MRI scores. *In vivo* AAV‐mediated gene therapy studies have been completed (Corti *et al*, 2017) or are in progress.

However, like for ERT, important challenges remain to be faced also by gene therapy, particularly the need for sustained expression of the therapeutic enzyme, and the need for correction of neuropathology. To address this latter issue, studies aimed at correcting brain involvement through direct intraparenchymal or intrathecal vector administration have been performed for the treatment of mucopolysaccharidose types IIIA and IIIB (Tardieu *et al*, 2014; Tardieu *et al*, 2017).

Other approaches based on gene editing for mucopolysaccharidosis types I and II (Data ref: ClinicalTrials.gov NCT03041324), inhibition of nonsense‐mediated decay and translational read‐through (Banning *et al*, 2018), and messenger RNA (mRNA) therapy (an approach based on biosynthetic mRNA transcripts to drive the synthesis of therapeutic proteins) (Zhu *et al*, 2019) are under evaluation.

Other strategies that are currently under investigation are based on the use of antisense oligonucleotides that allow for rescue of the normal splicing of transcripts. For example, this approach appears to particularly attractive for the treatment of late‐onset Pompe disease patients, in which a c.‐32‐13T > C mutation causes aberrant splicing and exon 2 partial or complete skipping with reduced synthesis of normal mRNA. This variant is highly prevalent (40–70% of alleles). Thus, a therapy for this mutation would have the advantage of being effective in a large fraction of patients. The characterization of splicing regulatory elements in GAA intron 1 and exon 2 and of the effects of the c.510C > T variant that modulates the effects of the c.‐32‐13T > C mutation (Bergsma *et al*, 2019) helped developing an antisense oligonucleotide‐based approach to promote exon 2 inclusion and enhanced GAA enzyme activity to levels above the disease threshold (van der Wal, 2017). Preliminary *in vitro* data show promising rescue of acid alfa glucosidase activity in cells from Pompe patients by using this approach (Goina *et al*, 2017; van der Wal, 2017).

### Adjuvant therapies

In view of the emerging complexity of LSD pathophysiology, novel strategies are being explored that are based on an entirely different rationale and may represent adjunctive approaches to the treatment of LSDs. These approaches are not directed toward correction of the enzymatic defects and the causative gene mutations, or on modulation of the flux of substrates to lysosomes, but they are rather targeted to the manipulation of the pathways that are secondarily altered in LSDs.

For example, the abnormalities of the autophagic pathway are attractive targets. Indeed, manipulation of this pathway has been proposed as a therapeutic strategy for Pompe disease, providing some evidence of efficacy. *In vitro* and *in vivo* overexpression of TFEB also induced exocytosis, enhanced glycogen clearance, and resulted in some improvements in physical performance of Pompe disease mice (Spampanato *et al*, 2013; Gatto *et al*, 2017). *In vitro* overexpression of TFE3 triggered lysosomal exocytosis and resulted in efficient cellular clearance (Martina *et al*, 2014). This approach that has been explored so far through overexpression of master genes controlling the autophagic pathway may be difficult to translate into clinical applications. However, based on these proof‐of‐concept studies, alternative strategies based on the search for small‐molecule drugs modulating autophagy may be envisaged, with a better potential for clinical translation.

Aberrant activation inflammation is another potential therapeutic target. For example, as neuroinflammation has been documented in several LSDs, the pathways that are involved in the activation of the inflammasome are now being considered as additional potential therapeutic targets. Pentosan polysulfate, a mixture of semisynthetic sulfated polyanions, has been shown to have anti‐inflammatory effects in some LSDs, in particular targeting the activation TLR4 and the consequent secretion of proinflammatory cytokines and tumor necrosis factor (TNF)‐α. This drug has been tested in mucopolysaccharidosis type I and II patients and in animal models of mucopolysaccharidosis types I, IIIA, and VI (Simonaro *et al*, 2010; Simonaro *et al*, 2016; Orii *et al*, 2019), and in *in vitro* models of Fabry and Gaucher disease (Crivaro *et al*, 2019). Intraperitoneal high‐dose aspirin reduced neuroinflammation in mucopolysaccharidosis type IIIB mice, with significantly reduced transcript levels of MIP‐1α, IL‐1β, and GFAP (Arfi *et al*, 2011). A combination of Miglustat as a substrate‐reducing agent, the Ca^2+^‐modulator curcumin, and a non‐steroidal anti‐inflammatory drug to target neuroinflammation was evaluated in Niemann‐Pick disease type C1 mice and resulted into maintained body weight and motor function, reduced microglial activation, and delayed onset of Purkinje cell loss (Williams *et al*, 2014).

Other experimental therapeutic approaches have been directed toward correction of intralysosomal calcium levels in Niemann‐Pick disease type C1 (Lloyd‐Evans *et al*, 2008) and reduction in oxidative stress in Krabbe disease (Hawkins‐Salsbury *et al*, 2012), while stimulation of the cytoprotective effect of HSP70 with the small‐molecule arimoclomol in Niemann‐Pick disease type C1 is under clinical evaluation (Kirkegaard *et al*, 2016).

Although these approaches directed toward correction of the secondary abnormalities in LSDs are not expected to be curative, they may be of help in improving quality of life and slow disease progression. It is possible to speculate that correction of these abnormalities may synergize with existing therapies. For example, in Pompe disease the block of autophagy has been shown to impact on the lysosomal trafficking of the recombinant enzyme used for ERT (Fukuda *et al*, 2006b). It may be conceivable that improving the status of autophagic pathways may translate into improved lysosomal delivery of the therapeutic enzyme.

It is possible that other potential therapeutic targets will be identified thanks to the precise characterization of the pathogenetic cascade of LSDs and will open new avenues to the treatment of LSDs.

## Conflict of interest

A. Ballabio is co‐founder of CASMA Therapeutics and of Next Generation Diagnostics (NGD).

**For more information**

- NORD—National Association for rare diseases: https://rarediseases.org/rare‐diseases/lysosomal‐storage‐disorders/

- Lysosomal Disease Network Patients & Families: https://lysosomaldiseasenetwork.org/

- National Gaucher Foundation: https://www.gaucherdisease.org/

- National Fabry Disease Foundation: https://www.fabrydisease.org/

- Sanfilippo Children Association: https://www.sanfilippo.org.au/

- Acid Maltase Deficiency Association: https://amda‐pompe.org/

- European Reference Network for Hereditary Metabolic Disorders: https://metab.ern‐net.eu/

- Tigem—The Telethon Institute of Genetics and Medicine: https://www.tigem.it/

- Telethon Foundation: https://www.telethon.it/

**Pending Issues**

The current understanding of lysosome biology and function is still evolving. There is a need for further characterization of these aspects that may provide critical information on the pathophysiology of lysosomal storage diseases.

New methodologies should be exploited to improve our knowledge on lysosomal biology and on lysosomal disease pathophysiology. These methodologies may also have a major impact of patient care, with more efficient diagnostic pathways and availability of biomarkers to follow disease progression and effects of therapies.

Current therapies for the treatment of lysosomal storage disease have significant limitations. Particularly, biodistribution in target organs, such as brain, is a critical issue as many of these disorders are associated with central nervous system involvement.

The understanding of disease pathophysiology is critical as it has the potential to identify novel therapeutic targets and to indicate new strategies for the treatment of these disorders.
